# Critical perspectives on Arts on Prescription

**DOI:** 10.1177/17579139231170776

**Published:** 2023-05-12

**Authors:** H Bungay, A Jensen, N Holt

**Affiliations:** School of Allied and Public Health, Anglia Ruskin University, East Road, Cambridge CB1 1PT, UK; Primary Healthcare, Lund University, Lund, Sweden; Aalborg University, Aalborg, Denmark; Department of Health and Social Sciences, University of the West of England, Bristol, UK

**Keywords:** Arts on Prescription, mental health and wellbeing, social prescribing, primary healthcare, referrals

## Abstract

The positive outcomes of engaging in the arts are increasingly reported in the research literature, supporting the use of the arts to enhance individual and community health and wellbeing. However, little attention is given to the less positive aspects of arts engagement. In some countries, healthcare practitioners and link workers can refer service-users experiencing mental health issues to social interventions such as Arts on Prescription (AoP) programmes. This critical review identifies problematic issues across such social prescriptions and AoP, including failures in arts and health projects, participants’ negative experiences, and an absence of ethical guidelines for arts and health practice. Furthermore, it is evident that there is a lack of awareness and knowledge within healthcare systems, leading to inappropriate referrals, failure to take account of individual preferences, and a lack of communication between the third sector and healthcare services. Significantly, it is also unclear who holds the health responsibility for AoP participants. This article raises more questions than it answers, but for AoP to be effectively embedded in healthcare practice, the issues highlighted need to be addressed in order to safeguard participants and support the effective implementation of programmes more widely.

## Introduction

In the arts and health field, we have for the past two decades been busy gathering and presenting data supporting positive outcomes of arts participation for health and wellbeing.^1^ However, little attention has been given to the less encouraging aspects of the field, including failures in arts and health projects, the negative experiences of some participants, or difficulties embedding arts for health within healthcare systems.^2^ Since Arts on Prescription (AoP) programmes are increasingly recommended by research and policy,^3–7^ this review presents a much-needed critical perspective on the current evidence base and practice.

Our focus is on AoP, which falls under the umbrella of social prescribing (SP).^8–10^ SP is a way for primary healthcare professionals, such as General Practitioners (GPs) and allied health professionals, to refer service-users to community-led activities (including gardening, cooking, walking, creative activity, or other groups), with the expectation that this will improve psychosocial wellbeing).^8,9^ AoP involves referral to a programme of arts activities, primarily offered to service-users experiencing stress, anxiety, mild to moderate depression and social isolation.^3,6,10^ AoP programmes can consist of participating in different arts activities (e.g. painting, sculpting, visiting museums) in small groups, facilitated by an artist or an arts educator. Sessions are typically held once or twice weekly and last for 2 h, while programmes vary in length, from 6 to 12 weeks.^6–8,10^ AoP differs from art therapy since art is not being used to facilitate psychotherapy or emotional expression. The focus is on process and play, rather than skill development, and the facilitator works to create a ‘safe space’ where this can occur.^4,5^ There is wide diversity in the structure and delivery of AoP programmes, including different names and models of operation, referral processes, target populations and funding methods.^7,8,10^

While participation in art activities can promote mental health and wellbeing, alleviate symptoms of stress, anxiety, and depression, and improve outcomes for those diagnosed with serious mental health illness, when provided as a supplement to other interventions,^1,3,6–8^ we are also aware that arts participation can have little impact on wellbeing, or even negative outcomes.^11–16^ Further, while it has been argued that AoP, and SP more widely, have the potential to alleviate pressures on the healthcare system (e.g. by reducing GP visits)^9^ they have also created problems of a structural and ethical nature.^2^ We want to challenge the assumption that AoP is always without negative effect, in order to better recognise, acknowledge, and learn from practice and critical observations in the existing literature. In this article, we will focus on what we understand as pertinent issues, drawing on research and on our own experiences as researchers in the arts and health field, considering both the structures through which service users are referred, and the roles of individual facilitators and organisations delivering arts programmes.

## Literature Review

We conducted a search of the literature using the terms ‘art on prescription’, ‘arts on prescription’, ‘art on referral’, ‘arts on referral’, ‘social prescribing’, ‘art and health’ in the research databases; Science Direct, PsychINFO, Primo, Ebscohost, Web of Science, PubMed, Design and Applied Arts Index, and the Cochrane Library. We limited our search dates from 1994 (when the first art on prescription programme was delivered) to 2021 and included an Internet search for grey literature. We reviewed papers and reports for limitations, weaknesses and critical commentary. Through notes, annotated bibliographies and discussions, this led to the identification of practical and structural challenges with the delivery of AoP as well as gaps in the existing evidence base. As a result we identified specific topics and in this paper will explore: (1) structural problems with embedding AoP within health systems, including: referrer’s knowledge about AoP; health responsibility (how this is managed across the stages of the referral process and beyond), and how feedback is given to referrers and (2) Challenges and barriers for participants and artist facilitators, including next-step opportunities for participants, group dynamics in AoP workshops, and training opportunities for artists facilitators.

## Referrer’s Knowledge about AoP

There is little published research specifically about healthcare practitioners’ knowledge and understanding of AoP.^17,18^ Yet, the research literature on SP is rapidly expanding and the issues experienced in SP are similar to AoP, as they both provide adjunct social activities for service-users, usually provided by third sector organisations, with the potential to remove pressure from statutory services. However, to be effective, referrals to different services and activities need to meet the needs of those referred. Variations in services can be problematic, with a lack of knowledge and understanding of a service leading to inappropriate referrals.^19^ It can be difficult for primary healthcare providers (such as GPs, Link Workers and others who play a ‘navigating role’ in SP, connecting people with community activities) to be aware of services provided by the voluntary sector and to remain up to date with activities available in a particular area.^2,19^ Primary healthcare providers may refer service-users to AoP programmes because they want to offer them ‘something’ rather than nothing,^17^ but there is also the potential for GPs to ‘offload’ service-users who are seen to be difficult or who require more intensive support than AoP is designed to provide.^18,20,21^ Indeed, a study of SP link workers’ experiences reported concern about referrals of service-users with severe mental health problems, including psychosis and suicidal ideation,^20^ which link workers and community partners did not have sufficient training to work with confidently, and for whom aspects of the intervention (such as completing evaluation forms) led to distress, and to distress of other group members (e.g. concern over disclosure of suicidal thoughts). It is therefore important that referrers not only understand both benefits and potential harms of the activities offered but also their suitability for the person being referred.^21^

Inappropriate referrals are not a benign issue, staff delivering the activities may lack capacity and the necessary expertise to support people with complex mental and physical health needs.^21^ In such situations, it is not just the staff and the service-users who may suffer, but the experience of the whole group may be compromised. This is not to say that programmes for such groups could not be devised, however, it becomes problematic if AoP programmes are used without sustainable plans for the service-users, including careful consideration of the appropriateness of programmes. This requires collaboration between those who make referrals and activity providers, which also raises the question about health responsibility.

## Who Holds Health Responsibility?

There are a variety of different stakeholders promoting and delivering AoP activities. Service-users can progress along a ‘referral pathway’, for example, from a GP to a link worker, to a community group led by an artist facilitator.^8,9^ If activities are delivered within a framework promoting mental health and wellbeing – or are offered as a prescription from a healthcare provider – then there is a health responsibility and duty of care to consider.^22,23^ This has been emphasised by cultural institutions delivering AoP activities who appreciated the presence of an AoP-coordinator to hold the health responsibility for the group so they could concentrate on facilitating arts activities.^24^ Yet, this is an area within arts and health practice that remains unclear.^23^ The commitment given by the referrer to work with a service-user varies across programmes, meaning that not all individuals referred are monitored by the primary care network across AoP programmes.^19^ This issue is especially pressing, since, while there is promising evidence of the positive value of arts engagement,^3–8^ there are also examples where participation has led to harm, such as re-living traumatic experiences,^25^ and the end of a programme can trigger feelings of loss and despair.^14^

In medical practice, an essential principle is to ‘do no harm’.^23^ This principle should likewise apply to arts for health activities, and stakeholders have a responsibility to ensure no harm for participants.^23^ This is not to say that arts participation should be without challenge; certain levels of stimulating and thought-provoking engagement are positive and acknowledged as reasons for why the arts can contribute to a healing process. However, it is crucial that someone holds the health responsibility for the referred individual, monitoring wellbeing during a programme.

## Feedback on Service-Users/Follow Up

Lack of information about referral schemes, uncertainty about service-user’s eligibility for a programme or the nature of the activity, combined with a lack of feedback about the service-users’ progress, have been perceived to be major barriers for GPs to engage with an Exercise on Referral Scheme.^26^ Similarly, recent unpublished research by the authors found evidence that referring practitioners rarely received feedback on the people they had referred to AoP programmes. This was further identified as an issue for SP, with link workers not receiving feedback from organisations where they had referred people.^27^ It can be difficult for primary care practitioners to keep track of people referred to activities under the umbrella of SP because many of the activities are provided by the voluntary sector with no formal mechanisms in place for follow-up and lack of infrastructure for tracking. Yet GPs have been reported to perceive regular feedback about how service-users were doing in a SP programme to be important^2^ and wanted to see more evidence for the effectiveness of the specific SP programmes they referred service-users to.

## Next Step Opportunities

In line with absence of follow-up by practitioners, we are also aware that often there are no next-step opportunities for participants,^17,18^ which can create fear and distress for the participants at the end of a programme.^14^ Lack of new pathways leaves some participants facing a void when activities come to an end and can create anxiety for participants who can no longer attend the group.^4,14^ Participants who have experienced mental health benefits and gained motivation are often left without prospects of next step opportunities and no real options for further progression.^7^ To retain the motivation and level of wellbeing associated with participation in AoP, being able to provide follow-up after the group sessions and transitions to other initiatives, is required.^3^ AoP can act as catalysts or as stepping stones for participants.^5^ However, to further self-development and provide progression pathways, necessitates a platform with continuous opportunities as well as an overarching coordinated plan, which demands collaboration between different health and social sector stakeholders (and the third sector).

## Group Dynamics

Beyond considering the challenges related to structures and health responsibility it is necessary to also reflect on the nature of the AoP sessions themselves. Participants in AoP programmes consistently emphasise the importance of the ‘group’ for individual wellbeing, both in terms of the support found from peers and the care and understanding provided by the facilitator.^4,5,14,28^ This occurs through the creation of a ‘safe space’ by the facilitator, where play and exploration are enabled and stigma and judgement withheld.^4,5^ One potential mechanism by which AoP improves wellbeing, in this space, is through social bonding, where people make meaningful, supportive connections with each other, as part of a ‘social cure approach’.^14,29^

Given the importance of social bonding, inadequate attention has been given to the converse: what happens when social bonding does not occur; or when the group space is not perceived as safe?^30,31^ In a systematic review of participatory arts for wellbeing it was found that some participants experienced not being part of the group, and stigmas of exclusion were consequently reinforced, increasing feelings of isolation and negatively impacting self-worth.^14^ Wellbeing interventions that seek to develop social bonding should be aware of the ‘dark side’ of social capital, and seek to reduce the risk of adverse outcomes.^31^ For example, being aware that some individuals may find engaging in shared practices difficult (e.g. experiencing distress or embarrassment), pressure to complete tasks stressful or the emotional labour of supporting others burdensome.^14,30,31^ Some communities may need longer to build trust, some practices might reinforce social divisions (based on class, ethnicity of gender), and some group norms may be ‘unhealthy’ (e.g. identifying as depressed).^1,14,30,31^

Research in this area is limited since the voices of those who have not formed social relationships with the group may not be captured in research, due to selection biases with data collection and attrition rates, where these individuals may be less likely to reach the end of a programme, when such data are typically collected. For example, Crone et al.^32^ noted that about one third of participants drop out of AoP programmes (and these were most likely to be referrals with the lowest wellbeing scores, who may need additional support to engage with programmes).

## Inconsistent Practice

The work of facilitators and the safe space that they help to create in art sessions has been described as critical to the success of AoP groups.^33^ However, there is little research as to how artist facilitators create this safe space, and little training for practitioners, new and experienced, to help learn and develop these skills.^18,34^ There is a long-standing yet growing awareness of the need for more support and training for AoP artists and generally for arts practitioners working in health.^33^ Furthermore, there is an identified need for staff at cultural institutions to be equipped with skills to support inclusive ways for working with vulnerable people.^18^ As AoP expands, it is imperative that artists (and cultural staff) are adequately prepared to work with vulnerable people in different settings. Currently, there is no consistent training for practitioners in essential areas such as safeguarding, ethics, evaluation, equal opportunities, data protection, health and safety, confidentiality policy, communication in healthcare settings, or the health needs of specific groups.^34^

## Future Directions

Based on the issues identified above there are numerous implications for policy makers, service providers, and practitioners to consider when including AoP and other similar social initiatives in care pathways. This is especially pertinent since there is no statutory regulation of AoP services. These include the provision of training for referring practitioners, clarification of where health responsibilities are placed once service-users are referred to an intervention or activity outside of the statutory services, the establishment of networks of communication and feedback between stakeholders, and training and guidelines for those who facilitate the activities.

## Implications for Primary Care

Those working in healthcare require training in how to engage community groups to support SP and obtain knowledge of the evidence for such activities.^2^ However, achieving this may be difficult, particularly considering findings that, although GPs admitted to a lack of awareness of non-medical sources of support, they did not see it as their responsibility to identify such sources of support for their service-users.^35^ There are different requirements of professionals who are involved in SP and AoP models, for example, link workers who need awareness and sensitivity to the specific context, communities, and characteristics of participants.^36^ Researchers have recommended that GP practices provide information and training for *all* employees about the remit and role of SP processes.^22^ This is particularly relevant in the UK as increasing numbers of GP practices are employing link workers whose effectiveness could be limited by a lack of training and knowledge of local social activity opportunities for service users.^2,8^

One way to provide continuity and transparency for healthcare providers and the individual service user would be by establishing networks between partners in the delivery of AoP (to include health, social care, and third sector organisations such as community arts) that could share information and coordinate good practice.^37^ Such networks could co-produce and design services to meet local need, (e.g. groups for specific health needs or demographics), streamline evaluation, adapt delivery design, and provide feedback to health practitioners, about the success (or otherwise) of AoP programmes for individual service-users. Collaborations and feedback between health and community-based art organisations that provide activities for health and wellbeing have the potential to decrease cross-cultural differences, for example, in language use and values, and increase effective engagement across different sectors, such as involvement in commissioning negotiations.^38^

## Responding to the Duty of Care

The question of health responsibility raises many more questions, including that of service-user individual responsibility and the wider aspect of society’s obligation for health of its citizens. Assuming that society is partly responsible for the health of its members, does not answer the question as to how this responsibility can be met.

Duty of care includes acknowledging and safeguarding against potential risks of AoP participation.^23,31^ For example, referrers’ sensitivity to the specific context, communities, and characteristics of participants, considering individual identities and vulnerabilities is essential for appropriate referrals.^19,30^ Facilitators could be supported to develop group identification, considering optimal group sizes, whether to embed socialising opportunities into the programme, working out how to best identify those who appear to feel excluded and how to manage this and extend in-group support to all members (e.g. by reinforcing inter-group commonalities, such as a shared identity of artist).^14,30^ Providing resources to train staff and enable the monitoring of service-users across programmes is essential to maintain the duty of care.

## Ethics and Guidelines for Artists and Practitioners

While individual styles are a necessary part of complex interventions, and artistic freedom an important aspect of participatory arts activities, there is nevertheless a problematic lack of consistency with approaches across different AoP programmes, in part brought about by a lack of training and sharing of good practice, but also because evaluation and research is primarily focused on participants rather than facilitators and delivery.^18,39^ There is a lack of transparency and documentation regarding the role of art facilitators in AoP practice and an absence of both good practice guidelines and an ethical code of practice, which go hand-in-hand and are essential for professional practice.^24,39^ Over the years, there have been several attempts in the English-speaking countries to develop guidelines for arts and health^39^ and most recently by the National Organisation for Arts in Health (NOAH) in the US.^40^ Codes of practice for artists and facilitators could include guidance on the maintenance of personal boundaries, personal disclosures, management of interpersonal issues in the groups, and sensitivity and responsivity to individual needs, as well as embedding peer supervision that sustains practitioner wellbeing and professional practice. Such guidelines would contribute to improving standards, critical thinking and strategic planning, not only for AoP programmes but also in the wider arts and health field.

## Conclusion

This article has perhaps raised more questions than it has answered. As practitioners and researchers, we encounter a variety of different ethical dilemmas. For example: when to intervene if we do not know the group of participants well? When should healthcare professionals be included as a function of support? How can conflicts that may arise in a group dynamic be managed? Is it acceptable not to have any ‘next step’ options to offer participants? These are some of the questions that we can regularly ask ourselves, and the answers may be ambiguous. However, training and ethics, and good practice guidelines would be able to assist in difficult situations. The larger question of who holds the health responsibility in a healthcare system, with different (and new) stakeholders delivering arts for health initiatives and interventions, remains unanswered.

However, consideration of implications for AoP as a healthcare practice is required at different stages of the AoP-model: primary healthcare practitioners have essential functions in identifying the need for referral, the link worker/coordinator in identifying the appropriate group and preferences of the individual, the artist facilitator/coordinator in managing the AoP group, and the link worker/healthcare practitioner in supporting transitions when groups are coming to an end, through re-referrals, referral to move-on groups, or peer-led groups. It is necessary to better understand the structures and the gaps in connecting all stakeholders. Therefore, to provide a solid foundation for policy it is necessary to encourage more extensive research so policymakers can reach relevant decisions. Furthermore, it is appropriate to develop guidelines and codes of conduct to support and professionalise art and health practices. While it is over a decade since White^39^ first introduced the concept of ethical and practice guidelines, we highlight the continued need for such guidelines to protect both participants, practitioners, and researchers, and we suggest that the task of developing these is done in consultation with all stakeholders. If AoP and other art and health practice are to become an integral part of health promotion, treatment, and rehabilitation, some level of formalisation of the field of practice is undoubtedly necessary. Therefore, we conclude that the development of guidelines should be considered a priority.

## References

[1] Tomlinson A Lane J Julier J , et al. A systematic review of the subjective wellbeing outcomes of engaging with visual arts for adults ( ‘working-age’, 15-64 years) with diagnosed mental health conditions. Report, What Works for Wellbeing, London, 2018.

[2] Aughterson H Baxter L Fancourt D . Social prescribing for individuals with mental health problems: a qualitative study of barriers and enablers experienced by general practitioners. BMC Fam Pract 2020;21(1):194. 32957923 10.1186/s12875-020-01264-0 PMC7507286

[3] Sumner RC Crone DM Baker C , et al. Factors associated with attendance, engagement and wellbeing change in an arts on prescription intervention. J Public Health 2020;42(1):e88–95. 10.1093/pubmed/fdz032 30957172

[4] Hughes S Crone DM Sumner RC , et al. Understanding well-being outcomes in primary care arts on referral interventions: a mixed method study. Eur J Pers Center Health 2019;7(3):1768.

[5] Stickley T Hui A . Social prescribing through arts on prescription in a UK city: participants’ perspectives (part 1). Public Health 2012;126(7):574–9. 10.1016/j.puhe.2012.04.002 22683358

[6] Holt NJ . Tracking momentary experience in the evaluation of arts-on-prescription services: using mood changes during art workshops to predict global wellbeing change. Perspect Public Health 2020;140(5):270–6. 10.1177/1757913920913060 PMC7498906 32441226

[7] Jensen A . Culture vitamins – an arts on prescription project in Denmark. Perspect Public Health 2019;139(3):131–6. 10.1177/1757913919836145 30955476

[8] Chatterjee H Polley MJ Clayton G . Social prescribing: community-based referral in public health. Perspect Public Health 2017;138(1):18–9. 10.1177/1757913917736661 29290159

[9] Bickerdike L Booth A Wilson PM , et al. Social prescribing: less rhetoric and more reality. A systematic review of the evidence. BMJ Open 2017;7(4):e013384. 10.1136/bmjopen-2016-013384 PMC5558801 28389486

[10] Bungay H Clift S . Arts on prescription: a review of practice in the UK. Perspect Public Health 2010;130(6):277–81. 10.1177/1757913910384050 21213564

[11] Daykin N Mansfield L Meads C , et al. The role of social capital in participatory arts for wellbeing: findings from a qualitative systematic review. Arts Health 2021;13(2):134–57. 10.1080/17533015.2020.1802605 32809907

[12] Heaslip V Darvill T . Human henge wellbeing research. Report, Bournemouth University, Bournemouth, 2018.

[13] Elkins N Bailey J . Evaluation of the VicHealth Active Arts Integration 2017-2018. New York: BYP Group; 2018.

[14] Daykin N de Viggiani N Moriarty Y , et al. Music-making for health and wellbeing in youth justice settings: mediated affordances and the impact of context and social relations. Sociol Health Illn 2017;39(6):941–58. 10.1111/1467-9566.12549 28332197

[15] Southcott J Nethsinghe R . Resilient senior Russian-Australian voices: ‘we live to sing and sing to live’. Appl Res Qual Life 2019;14(1):39–58.

[16] Boydell KM . Making sense of collective events: the co-creation of a research-based dance. Forum Qual Soc Res 2011;12(1):1525.

[17] Stickley T Hui A . Social prescribing through arts on prescription in a UK city, referrers’ perspective (part 2). J Public Heal 2012;126(7):580–6. 10.1016/j.puhe.2012.04.001 22578297

[18] Jensen A Bungay H . Swedish primary healthcare practitioners’ perspectives on the impact of arts on prescription for patients and the wider society: a qualitative interview study. BMC Health Serv Res 2021;21(1):1277. 34836515 10.1186/s12913-021-07258-7 PMC8626132

[19] Sandhu S Lian T Drake C , et al. Intervention components of link worker social prescribing programmes: a scoping review. Health Soc Care Community 2022;30(6):e3761–74. 10.1111/hsc.14056 36181384

[20] Hazeldine E Gowan G Wigglesworth R , et al. Link worker perspectives of early implementation of social prescribing: a ‘Researcher-in-Residence’ study. Health Soc Care Community 2021;29(6):1844–51. 10.1111/hsc.13295 33528060

[21] Wildman JM Moffatt S Penn L , et al. Link workers’ perspectives on factors enabling and preventing client engagement with social prescribing. Health Soc Care Community 2019;27(4):991–8. 10.1111/hsc.12716 30637826

[22] Brown RCH Mahtani K Turk A , et al. Social prescribing in national health service primary care: what are the ethical considerations? Milbank Q 2021;99(3):610–28. 10.1111/1468-0009.12516 PMC8452361 34170055

[23] Jensen A . ‘First, do no harm’ in arts and health practices. J Appl Art Heal 2014;5(3):331–9.

[24] Jensen A Bonde LO . An arts on prescription programme: perspectives of the cultural institutions. Community Ment Health J 2020;56(8):1473–9. 10.1007/s10597-020-00591-x 32100154

[25] Vukcˇević Marković M Bjekicć J Priebe S . Effectiveness of expressive writing in the reduction of psychological distress during the COVID-19 pandemic: a randomized controlled trial. Front Psychol 2020;11:587282. 33240180 10.3389/fpsyg.2020.587282 PMC7683413

[26] Din NU Moore GF Murphy S , et al. Health professionals’ perspectives on exercise referral and physical activity promotion in primary care: findings from a process evaluation of the National Exercise Referral Scheme in Wales. Health Educ J 2015;74(6):743–57. 10.1177/0017896914559785 PMC4604423 26527835

[27] Runacres J . Social prescribing in practice: a critical examination of service data and stakeholder perspectives. Birmingham: Birmingham City University; 2019.

[28] Poulos RG Marwood S Harkin D , et al. Arts on prescription for community-dwelling older people with a range of health and wellness needs. Health Soc Care Community 2019;27(2):483–92. 10.1111/hsc.12669 PMC7379368 30345578

[29] Williams E Dingle GA Jetten J , et al. Identification with arts-based groups improves mental wellbeing in adults with chronic mental health conditions. J Appl Soc Psychol 2019;49(1):15–26.

[30] Wakefield JRH Bowe M Kellezi B , et al. When groups help and when groups harm: origins, developments, and future directions of the ‘social cure’ perspective of group dynamics. Soc Personal Psychol Compass 2019;13(3):e12440.

[31] Villalonga-Olives E Kawachi I . The dark side of social capital: a systematic review of the negative health effects of social capital. Soc Sci Med 2017;194:105–27. 10.1016/j.socscimed.2017.10.020 29100136

[32] Crone DM Sumner RC Baker CM , et al. ‘Artlift’ arts-on-referral intervention in UK primary care: updated findings from an ongoing observational study. Eur J Public Health 2018;28(3):404–9. 10.1093/eurpub/cky021 29462307

[33] Moss H O’Neill D . What training do artists need to work in healthcare settings? Med Humanit 2009;35(2):101–5. 10.1136/jmh.2009.001792 23674706

[34] Baxter L Fancourt D . What are the barriers to, and enablers of, working with people with lived experience of mental illness amongst community and voluntary sector organisations? A qualitative study. PLoS ONE 2020;15(7):e0235334. 10.1371/journal.pone.0235334 PMC7332084 32614876

[35] White JM Cornish F Kerr S . Front-line perspectives on ‘joined-up’ working relationships: a qualitative study of social prescribing in the west of Scotland. Health Soc Care Community 2017;25(1):194–203. 26455723 10.1111/hsc.12290

[36] Shiell A Hawe P Kavanagh S . Evidence suggests a need to rethink social capital and social capital interventions. Soc Sci Med 2020;257:111930. 30219489 10.1016/j.socscimed.2018.09.006

[37] Southby K Gamsu M . Factors affecting general practice collaboration with voluntary and community sector organisations. Health Soc Care Community 2018;26(3):e360–9. 10.1111/hsc.12538 29327484

[38] Puebla Fortier J Coulter A . Creative cross-sectoral collaboration: a conceptual framework of factors influencing partnerships for arts, health and wellbeing. Public Health 2021;196:146–9. 10.1016/j.puhe.2021.05.017 34216813

[39] White M . Developing guidelines for good practice in participatory arts-in-health-care contexts. J Appl Arts Heal 2010;1(2):139–55.

[40] National Organization for Arts in Health (NOAH). Code of Ethics and Standards for Arts in Health Professionals, 2018. Available online at: https://thenoah.net/wp-content/uploads/2018/10/NOAH-Code-of-Ethics-and-Standards-for-Arts-in-Health-Professionals.pdf

